# Supplementary material for: Greensporone A, a Fungal Secondary Metabolite Suppressed Constitutively Activated AKT via ROS Generation and Induced Apoptosis in Leukemic Cell Lines
Source: Biomolecules. 2019 Mar 29;9(4):126. doi: 10.3390/biom9040126 (PMC6523683; doi:10.3390/biom9040126)
Supplement: Supplementary file 1 [file biomolecules-09-00126-s001.pdf]

Supplementary Figure S1

A

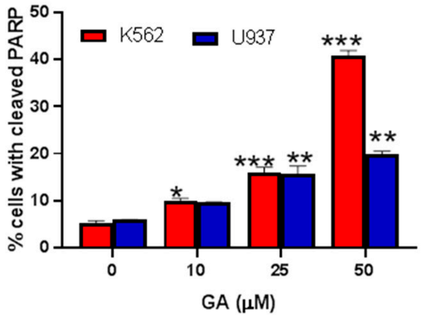

B

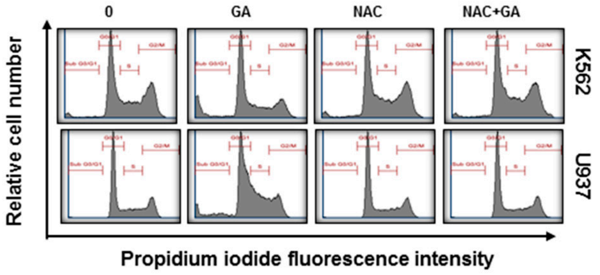

Supplementary Figure S1. **A.** GA induced PARP cleavage activity. K562 and U266 cells were treated with and without GA for 24 hours and levels of active cleaved PARP were determined by flow cytometry as described in Materials and Methods. The graph displays the mean +/- SD (standard deviation). **B. Pre-treated of leukemic cell with NAC prevent GA-induced SubG0 fractions**. K562 and U937 cells were pretreated with 10 mM NAC, subsequently treated with 25 μM GA as indicated for 24 hours. The cells were then analyzed by flow cytometry after PI staining.
